# Supplementary material for: What works best for ensuring treatment adherence. Lessons from a social support program for people treated for tuberculosis in Ukraine
Source: PLoS One. 2019 Aug 26;14(8):e0221688. doi: 10.1371/journal.pone.0221688 (PMC6709888; doi:10.1371/journal.pone.0221688)
Supplement: Attachment — Submitted filename: Response to reviewers.docx [file pone.0221688.s001.docx]

We provide our response to comments below in italic.

**Response to editors**

1. When submitting your revision, we need you to address these additional requirements.

Please ensure that your manuscript meets PLOS ONE's style requirements, including those for file naming. The PLOS ONE style templates can be found at
<http://www.journals.plos.org/plosone/s/file?id=wjVg/PLOSOne_formatting_sample_main_body.pdf> and <http://www.journals.plos.org/plosone/s/file?id=ba62/PLOSOne_formatting_sample_title_authors_affiliations.pdf>

*We followed the instructions in the templates and made revisions in the manuscript to meet PLOC ONE’s style requirements.*

2. Please provide additional details regarding participant consent. In the ethics statement in the Methods and online submission information, please ensure that you have specified (1) whether consent was informed and (2) what type you obtained (for instance, written or verbal). If your study included minors, state whether you obtained consent from parents or guardians. If the need for consent was waived by the ethics committee, please include this information

*We added details regarding participant consent (line 137-138 in the manuscript with tracked changes) and stated the following: “We informed participants of the study aims, risks and benefits for participation in the study, and obtained verbal informed consent prior to the interview.”*

3. We note that you have indicated that data from this study are available upon request. PLOS only allows data to be available upon request if there are legal or ethical restrictions on sharing data publicly. For more information on unacceptable data access restrictions, please see <http://journals.plos.org/plosone/s/data-availability#loc-unacceptable-data-access-restrictions>.

In your revised cover letter, please address the following prompts:

a) If there are ethical or legal restrictions on sharing a de-identified data set, please explain them in detail (e.g., data contain potentially sensitive information, data are owned by a third-party organization, etc.) and who has imposed them (e.g., an ethics committee). Please also provide contact information for a data access committee, ethics committee, or other institutional body to which data requests may be sent.

b) If there are no restrictions, please upload the minimal anonymized data set necessary to replicate your study findings as either Supporting Information files or to a stable, public repository and provide us with the relevant URLs, DOIs, or accession numbers. For a list of acceptable repositories, please see <http://journals.plos.org/plosone/s/data-availability#loc-recommended-repositories>.

We will update your Data Availability statement on your behalf to reflect the information you provide.

*We de-identified all transcripts from in-depth interviews with study respondents and made them available online from the University of North Carolina, Odum Dataverse repository.*

*This is the Data Availability Statement:*

*Data from the Ukraine Strengthening Tuberculosis Control Project Impact Evaluation – Phase 2 is available online from the University of North Carolina, Odum Dataverse repository (*[*https://dataverse.unc.edu/dataset.xhtml?persistentId=doi:10.15139/S3/MUSYTQ*](https://dataverse.unc.edu/dataset.xhtml?persistentId=doi:10.15139/S3/MUSYTQ)*) published in MEASURE Evaluation Dataverse (view at* [*https://dataverse.unc.edu/dataverse/MEP3*](https://dataverse.unc.edu/dataverse/MEP3)*). Anonymized transcripts are made available to researchers at no charge.*

**Response to reviewers**

Reviewers' comments:

Reviewer's Responses to Questions

**Comments to the Author**

1. Is the manuscript technically sound, and do the data support the conclusions?

The manuscript must describe a technically sound piece of scientific research with data that supports the conclusions. Experiments must have been conducted rigorously, with appropriate controls, replication, and sample sizes. The conclusions must be drawn appropriately based on the data presented.

Reviewer #1: Yes

Reviewer #2: Yes

*No response from the authors is required.*

2. Has the statistical analysis been performed appropriately and rigorously?

Reviewer #1: Yes

Reviewer #2: N/A

*No response from the authors is required.*

3. Have the authors made all data underlying the findings in their manuscript fully available?

The [PLOS Data policy](http://www.plosone.org/static/policies.action#sharing) requires authors to make all data underlying the findings described in their manuscript fully available without restriction, with rare exception (please refer to the Data Availability Statement in the manuscript PDF file). The data should be provided as part of the manuscript or its supporting information, or deposited to a public repository. For example, in addition to summary statistics, the data points behind means, medians and variance measures should be available. If there are restrictions on publicly sharing data—e.g. participant privacy or use of data from a third party—those must be specified.

Reviewer #1: Yes

Reviewer #2: Yes

*No response from the authors is required.*

4. Is the manuscript presented in an intelligible fashion and written in standard English?

PLOS ONE does not copyedit accepted manuscripts, so the language in submitted articles must be clear, correct, and unambiguous. Any typographical or grammatical errors should be corrected at revision, so please note any specific errors here.

Reviewer #1: Yes

Reviewer #2: Yes

*No response from the authors is required.*

5. Review Comments to the Author

Please use the space provided to explain your answers to the questions above. You may also include additional comments for the author, including concerns about dual publication, research ethics, or publication ethics. (Please upload your review as an attachment if it exceeds 20,000 characters)

Reviewer #1: A technically sound and academically well written manuscript. The qualitative data analysis was comprehensively explained. However, mention of inter rater reliability by comparing the coding and thematic analysis with co researchers and participants would have further strengthened the analysis. Nevertheless, the manuscript highlights relevant themes pertaining to adherence to TB treatment. Multiple drug resistant strains are a major public health concern of present times.

*Response: Thank you for your comment. We agree that calculation of inter-rater reliability would have further strengthened the analysis, however, we applied different techniques to validate the results of the analysis. Two researchers read all study transcripts to ensure that presented results reflect respondents’ views. The lead data analyst worked on coding and shared the code reports with another researcher for input. Both researchers reviewed the code reports, identified sub-themes in each code, and examined the evidence supporting the themes and sub-themes independently and then formed a consensus. Essential concepts and relationships between the different themes and sub-themes were formed. Both researchers worked on synthesizing data and presenting findings in the report.*

*To address this comment from the reviewer, we provided additional information in the analysis section of the manuscript.*

*This is the original version:*

*We reviewed the code reports, identified sub-themes in each code, and examined the evidence supporting the themes and sub-themes. Essential concepts and relationships between the different themes and sub-themes were formed. Data were synthesized**, and findings communicated through the process of writing up and presenting the data, using direct quotes to support the themes.*

*This is the modified version:*

*Two researchers read all study transcripts and reviewed the code reports, identified sub-themes in each code, examined the evidence supporting the themes and sub-themes independently and then formed a consensus. Essential concepts and relationships between the different themes and sub-themes were formed. Data were synthesized, and findings communicated through the process of writing up and presenting the data, using direct quotes to support the themes.*

Reviewer #2: The manuscript is written in tangible and organized way and touched critical area that should be addressed. However, some printing errors need revision. e.g. line 196, line 270

*Response: Thank you for your comment. We reviewed and revised the manuscript. We fixed errors in lines 196 and 270 as well as other errors related to translation of quotes from Ukrainian to English.*

6. PLOS authors have the option to publish the peer review history of their article ([what does this mean?](https://journals.plos.org/plosone/s/editorial-and-peer-review-process#loc-peer-review-history)). If published, this will include your full peer review and any attached files.


If you choose “no”, your identity will remain anonymous but your review may still be made public.


**Do you want your identity to be public for this peer review?** For information about this choice, including consent withdrawal, please see our [Privacy Policy](https://www.plos.org/privacy-policy).

Reviewer #1: No

Reviewer #2: No


*No response from the authors is required.*
